# Oxidative Coupling of Methane: A Review Study on the Catalytic Performance

**DOI:** 10.3390/molecules29194649

**Published:** 2024-09-30

**Authors:** Hamid Reza Godini, Madan Mohan Bhasin

**Affiliations:** 1 Chair of Process Dynamics and Operation, Technische Universität Berlin, Strasse des 17. Juni 135, 10623 Berlin, Germany; 2 Innovative Catalytic Solutions, LLC, 2048 Smith Road, Charleston, WV 25324, USA; bhasinmm2@gmail.com

**Keywords:** oxidative coupling of methane, catalytic performance analysis, catalyst characteristics, reaction engineering

## Abstract

Extensive data on the characteristics and performance of the catalysts synthesized and tested for methane oxidative coupling (OCM) is available in thousands of reports published during the last four decades. Revisiting and analyzing the general trends recognizable in those data could improve the current understanding of the catalyst functionality under different reaction conditions. This is instrumental in determining the direction of future research aiming for more efficient OCM catalysts and reactors. These are the subjects of the comprehensive analysis reported in this paper, which covers the main aspects associated with the analysis of the OCM catalytic performance, including the catalyst characteristics, reaction mechanism, and reactor operation. Special attention was devoted to analyzing these aspects in the framework of thermal-reaction engineering and, accordingly, critically reviewing the reported catalytic performances in the literature.

## 1. Introduction: Framing the Analysis Context

Oxidative Coupling of Methane (OCM) has the potential to evolve and become a competitive technology to at least partially address the ever-growing demand for ethylene production [1,2,3]. This has encouraged many researchers to investigate various aspects of this developing technology during the last four decades [4,5]. A large number of research studies have been justifiably devoted to catalyst synthesis and characterization as well as catalytic performance analysis under different reaction environments. These are the core issues yet to be fully understood by analyzing the reported data refined to distinctly represent the contributions of the catalyst functionality and the reactor’s operation. The extracted information should then be put together piece-by-piece to blueprint the OCM research in these areas and accentuate the development path forward. This could be, on the one hand, accomplished using a comprehensive analysis, relying on the verified understanding of the reaction mechanism and the phenomena involved in the catalyst surface shaping its kinetic performance. On the other hand, the contributions of the phenomena affecting the temperature and concentration profiles in the reactor scale and its selective safe performance should also be reflected in such analysis. This review paper is a step in that direction by setting the analysis framework and accordingly mapping and interpreting the so far reported data.

Ever since the beginning of the OCM research [6], developing a stable and selective catalyst has been the primary focus of the researchers. Subsequently, a wide range of aspects have been investigated primarily with regard to their impacts on stable and selective catalytic performance. These include the selection of catalysts’ materials and synthesis methods, as well as identifying the desired operating conditions in the reactor. On the other hand, the continuous advancement of the capabilities of characterization techniques has created new potential for a better understanding of the functioning mechanism of this high-temperature gas-phase catalytic reactions system.

The analysis starts with the general characteristics and functional features of OCM catalysts, particularly the research benchmark OCM catalysts with relatively distinguished stable selective performance. Methane and oxygen are, respectively, the excess reactant and limiting reactant, which is activated primarily on the surface of the catalyst as well as in the gas phase to produce ethane and, ultimately, ethylene. Heavier hydrocarbons, such as propane, propylene, etc., are also produced to a lesser extent. All hydrocarbons, including CH_4_, C_2_ (C_2_H_4_ and C_2_H_6_), and C_3+_ (C_3_H_6_ and C_3_H_8_ and heavier molecules), can undergo undesired conversions resulting in carbon oxides formation. Water and hydrogen are also generated as by-products in this reaction system.

The representative set of catalytic and gas phase reactions includes methane coupling (2, 13), methane partial and complete oxidation (1, 3, 10, 11), ethane thermal- and oxi-dehydrogenation (5, 12), water gas shift WGS (forward and reverse) reactions (8, 9) [7,8]. Ethylene, as the targeted product, can also be activated and transformed (6, 7, 14) to produce carbon oxides, hydrogen, and water in the gas phase and on the surface of the catalyst, where carbon monoxide can also be oxidized (4).

**Figure 1:**
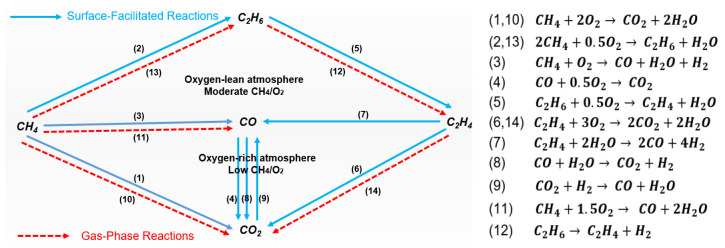
(**Left**) Schematic representation of the gas-surface OCM reactions network, (**Right**) main reactions involved.

A simplified schematic representation of the reactions network corresponding to the probable gas-surface reactions is shown in Figure 1 (adopted from reference [7]), not necessarily reflecting the weight or, for that matter, the dominance of any certain reaction path. Detailed information describing the gas phase and catalytic reaction paths comprising different sequences of the elementary and secondary reactions are explained elsewhere [7,8].

Depending on the targeted range of reaction temperature, the relative contributions of the catalytic-driven and non-catalytic gas-phase molecular reactions will be different so that the later ones will be intensified at a high temperature (>800 °C), whereas the contribution of the catalyst in determining the selectivity will be reduced [7,8,9,10,11,12]. The rate and contribution of each reaction under different sets of operation conditions are reflected in the main OCM reaction/reactor performance indicators, namely (a) Selectivity: portion of the converted methane ends up appearing in each specific product; (b) Yield: percentage of the inlet methane contributing in forming each specific product, (c) Methane Conversion: portion of the inlet methane converted to the desired and undesired products.

Similar to many other sets of parallel-series reactions, the reactant’s conversion and the desired product’s selectivity could not be simultaneously maximized, which limits the attainable yield of the products. For instance, methane conversion could be improved by lowering the methane-to-oxygen ratios. However, this not only results in a rapid reduction of selectivity but also the prospect of a safe operation of the OCM reactor, and therefore, the lower boundary of methane-to-oxygen ratio is restricted (e.g., CH_4_/O_2_ > 2). Therefore, no more than half of the inlet methane is expected to be converted by passing through the reactor, from which a significant portion is converted to carbon dioxide. Generating less carbon dioxide while converting a large portion of the methane in a single pass-through can be ensured using a selective catalyst, the right set of operating conditions, and a proper reactor design. A proper reactor design not only improves the selective conversion of methane by enabling the catalyst to perform close to its mechanistically desired conditions but also enables controlling the thermal behavior of this exothermic system. For instance, using a membrane reactor enables securing the desired temperature- and concentration profiles by keeping the local methane-to-oxygen ratio high along the catalytic bed, facilitating the selective conversion on the catalyst surface [13,14].

It should be highlighted that the net energy balance of the OCM reactions represents an exothermic fast reaction system. Methane activation and ethane dehydrogenation both on the catalyst surface and in the gas phase require significant bound-breaking energy and, therefore, are intensified at a high-temperature range (i.e., >800 °C). This has serious implications for the design and operation of OCM catalysts and reactors, as well as for interpreting the reported reactor performances so far. The majority of the OCM studies have been conducted in such a high-temperature range, and therefore, this review paper mostly reflects the observations in those studies. Much fewer references where OCM is under milder operating temperatures, even via photocatalytic conversion or alternative milder oxidants, have been reviewed [15,16] and are not covered in the current review analysis. The distinctive focus of the present paper is on explaining the OCM selectivity issues by tracking the local intensity of the generation of radicals, affected by the characteristics of the catalyst as well as the local concentration and temperature. This is discussed uniquely through the thermal-reaction analysis of this system.

## 2. Catalyst Functionality and Characteristics

In this section, the current perception of OCM catalyst functionality with regard to the impacts of the catalyst’s structural and material characteristics on its catalytic performance is reviewed and discussed. Special focus will be devoted to the research-benchmark OCM catalysts, their reported characteristics, and catalytic performances in light of the review of the OCM reaction mechanism and the gas and surface reactions involved. These need to be further clarified for analyzing and eventually tailoring the contributions of the support material and the active catalytic components to the performance of OCM catalysts. There is still no broadly accepted comprehensive understanding of these contributions in spite of extensive research conducted. To address this, utilizing advanced characterization techniques, particularly the ones capable of tracking the fast dynamic transformation of material phases and their interaction with the molecular and radical species under such a high-temperature reaction environment, could be instrumental [17,18,19,20,21,22]. Therefore, critically reviewing the current understanding of the catalyst functionality and refining it, primarily in light of the recently reported characterization data, is necessary for setting the aims and directions for future research.

### 2.1. Reaction Mechanism

#### 2.1.1. Mechanism-Study of the Activating Roles of Different Oxygen Species

The initiation steps are the relatively well-understood part of the catalytic OCM reaction mechanism. In these steps, as a result of the interaction of oxygen molecules and the catalyst surface, active oxygen species are formed, and methane gas molecules hitting the catalyst surface are thereby activated to generate methyl radials. Some of these radicals couple to form ethane molecules while thermally interacting with the catalyst surface. A smaller portion of the radicals may even contribute to forming longer hydrocarbons. The rest undergo further transformation while interacting with the oxygen species to form transient intermediates and, ultimately, the more stable carbon oxide molecules.

**Figure 2:**
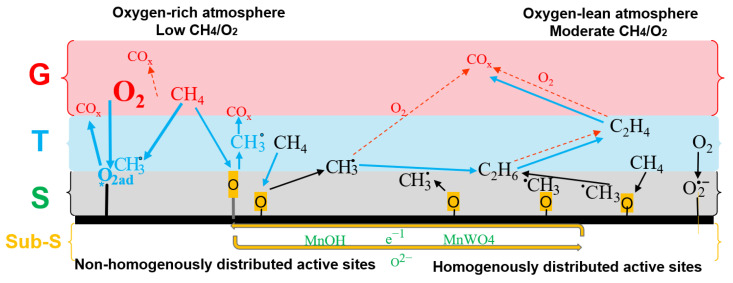
Schematic representation of the reactions pathways to follow the activation mechanism affected by the sub-surface, surface, and gas phase phenomena in the Gas (G), Transition (T), Surface (S) zones, Catalyst Subsurface (Sub-S); Predominant Unselective performance (Left-side); Predominant Selective performance (Right-side).

Following the involved species and analyzing the material characteristics of the OCM catalyst under reaction conditions enable tracking their involvement in the reaction mechanism and their impacts on selective conversion. This is crucial not only for identifying the desired characteristics of a selective OCM catalyst based on the consolidated understanding of the reaction mechanism but also for efficiently utilizing the catalyst under a proper reactor feeding and set of operating conditions [14,23,24,25,26,27]. In this context, distinct mechanisms under which different oxygen species get involved in the activation of methane and interact with the intermediate radicals and molecules have been particularly studied [19,27,28,29,30,31]. This enables tracking the selective and non-selective catalytic paths and ultimately correlating them with the desired catalyst characteristics. For instance, some reports have proposed and explained a relatively detailed mechanism in which the emphasis is on the distinct contributions of the adsorbed oxygen and lattice oxygen on the desired and undesired reactions [18,19,27]. However, not all aspects around the activating steps, and in general, the OCM reaction mechanism, are completely known or widely agreed upon. For instance, the individual and interactive catalytic contributions of the metal oxides in the activation of molecules and controlling the reactions-selectivity have not been entirely understood. Nevertheless, a schematic representation of the general deductive understanding of such a mechanism is shown in Figure 2 to serve as a base for further discussion and analysis. In this schematic, without discarding any possibility or claiming the dominance of any certain reaction path, a mechanism-superstructure is presented, in which the impacts of parameters could be tracked. Identifying three zones, respectively representing the catalyst surface (S), its vicinity transition (T) to farther distance in the gas phase (G), enables reflecting the distinguished impacts of different sets of catalyst characteristics and the involved phenomena on the activity and selectivity of the catalyst. The represented reaction paths are in accordance with the reaction network depicted in Figure 1. Detailed information about the reactions’ pathways could be found elsewhere [18,19,25,32,33,34,35,36].

In Figure 2, the gas phase components and reactions are shown in color red. The color grey represents the area close to the surface of the catalyst. The surface of the catalyst and all reactants being activated or generated through surface reactions have been colored black. The color light blue indicates the transition area between the surface area and gas phase area and the active primary components (surface saturated methyl radical and adsorbed oxygen) there. The dotted red arrows represent the gas phase reactions. The solid arrows show the surface (black arrows) or the surface-facilitated (blue-filled arrows) reactions. The bold or normal letters, respectively, demonstrate higher and lower concentrations.

As seen in Figure 2, the molecular oxygen isolated from the catalyst surface becomes involved primarily in the gas phase deep oxidation of ethylene and larger hydrocarbons owing to their relatively higher reactivity. Part of the adsorbed oxygen remaining on the catalyst surface in molecular form, particularly the one exposed to an oxygen-rich atmosphere (O_2_^2-^), is similarly prone to get directly involved in the oxidation of the active hydrocarbon species to carbon oxides. On one side, that oxygen becomes relatively easily accessible to the hydrocarbon molecules and radicals, which comprises a reactive atmosphere with an effective local low hydrocarbon-to-oxygen ratio close to the catalyst surface, pursuing a less selective activation-conversion path. On the other side, the oxygen dissociated on the catalyst surface generates active oxygen species in connection with the defects and lattice-oxygen-releasing capacity of the catalytic material. These can more selectively engage with the radicals in the vicinity of the catalyst surface to enhance coupling reactions. This could be emboldened when the activated oxygen and generated hydrocarbon radical species are distributed properly over the catalyst surface. Tracking the bulk- and spot-reaction intensity along the reactor enables examining this [7,9,33,37,38]. These will be explained further in detail in the next Section 2.1.2. Nevertheless, it could be summarized here that the oxygen over the catalyst surface becomes involved in generating methyl radicals primarily through the cycle of lattice oxygen generation-transfer-termination. The generated radicals can contribute favorably to forming ethane and ethylene through coupling reactions or undesirably react to form carbon oxides. The sequence of radical reactions contributing to the production of the later ones is expected to be dominating when the radicals are generated intensely and in an inhomogeneous flux rate over a surface spot. In this case, the on-spot generated radicals could oversaturate the surface, become detached from the surface, and actively engage in free-radical chemistry, tending towards the undesired gas phase radical reaction paths primarily prone to carbon monoxide formation.

These should be conceptually interpreted to understand the required characteristics of the catalyst and the reactor operation while accounting for the impacts of temperature as well as partial pressures of oxygen and methane, for instance. In this manner, for the oversaturated levels of the adsorbed or the locally activated oxygen over the catalyst surface, the intensity of the generated radicals exceeds the desired moderate generation rate, which could be further converted selectively there. Such a desired rate is determined in accordance with the available potential for selective catalytic conversion of the species, reflected through the homogeneously distributed concentration of the active sites and the reactive species. By exceeding the desired rate, non-selective conversion and undesired reactions intensify. The saturation capacity of the catalyst in this context is determined by the quantity and distribution of the active catalytic materials per volume or over the surface of the catalyst, affecting the local reaction intensity depending on the temperature and the local reactant concentrations. This is particularly consequential in the case of less homogenous distribution of the active catalytic materials, resulting in the spots with uneven strengths for interacting with the adsorbed oxygen or lattice oxygen across the catalyst surface. The inhomogeneity, in this case, is reflected in terms of the distance between the active spots as well as their activating strengths.

On the other hand, if the concentration of the oxygen interacting with the catalyst surface is lower than a certain threshold, so that the intensity of the generated radicals is too low, it also would harm the selective methane conversion because even if the active sites and oxygen species are well distributed across the catalyst surface, the chance for coupling reactions is reduced due to sub-optimal local rate of generation of methyl radicals and the frequency of their transverse interactions between the spots. In addition, if the active sites and oxygen species are poorly distributed across the catalyst surface, the intensity of the methyl radical generation in terms of concentration and rate could not be secured around its desired local value in most places. In both of these cases, the catalyst interaction with the reactive species will be sub-optimal, and it would be difficult to secure a significant amount of selective methane conversion, too. All in all, fine-tuning the parameters affecting the rates and distribution of methyl-radicals generation over the catalyst surface and along the bed secures a selective catalytic conversion. This is a key aspect in understanding the limitations and trade-offs in targeting and securing the highest achievable selective conversion.

#### 2.1.2. Catalyst Characterization to Track and Understand the Reaction Mechanism

Besides analyzing how the load and distribution of the active materials contribute to activating oxygen and methane, the impacts of the catalytic material’s characteristics in controlling the selective and non-selective conversion of species are also analyzed in this section, primarily for the reference catalysts. For instance, the extent and the mechanism through which manganese, sodium, and tungsten contribute to the selective performance of benchmark silica-supported Mn-Na-WOx catalysts should be further analyzed. In particular, it should be further analyzed how these components, which are close to their melting state under the OCM reaction temperature, enable homogenizing and moderating the transfer of lattice oxygen in the subsurface of the catalyst and thereby affect the reaction intensity and selectivity. As discussed earlier, tuning the local activation rate can affect catalytic selectivity and activity differently depending on the applied range of operating conditions.

On one side, a lower spot ratio of oxygen-to-methane results in low adsorbed oxygen in the catalyst scale, tuning down the cycle loop rate of the lattice-oxygen generation-transfer-termination beneath and around the catalyst surface. This consequently can tune the rate of generation of methyl radicals and improve the selectivity towards coupling reaction, as explained in Section 2.1.1 and depicted in Figure 2. This is conceptually in line with the suggested mechanism in some recent publications [19,25], yet not necessarily the exact way it has been interpreted there.

In fact, the need to establish a balance between the lattice oxygen supply and the adsorbed oxygen species highlights the selective-determining mechanism in this system. This results in a similar trade-off between conversion and selectivity, which is classically known for a wide range of parallel-series catalytic reaction systems. Having a fast catalyst, for instance, as a result of using relatively more active materials or using a larger load of them in the catalyst composition, does not overcome that trade-off. Rather, it shifts its position. This could partially explain how the material characteristics of each active component, for instance, ceria, known for its distinguished electron conductivity characteristics, can affect the rate of sub-surface lattice oxygen supply and the catalytic performance. Following a similar concept, it has been reported that low mobility of lattice oxygen in the catalyst support is advantageous for obtaining higher C_2_-selectivity [19,37].

On the other hand, the interactive impact of the spot temperature and the catalyst composition and characteristics on the selective and non-selective reactions should be investigated more systematically. The reported scattered observations could enrich such analysis, and therefore, such observations are reviewed here in this paper. For instance, it has been reported that the methyl radical generation over the catalyst surface is strongly temperature-dependent and is significantly slower than the temperature-independent radical coupling reaction [17,37]. The depicted mechanism in Figure 2 could be useful for explaining this by considering the rapid coupling of the well-distributed methyl radicals generated on the active sites. The slow generation of methyl radicals, though, might need to be facilitated using higher temperatures so that enough methyl radicals per area are generated to be mostly converted through coupling reaction.

In any case, only a limited portion of the generated methyl radicals get further converted through the coupling path for C_2_ production. The remaining part of the generated radicals over the catalyst surface does not statistically get the chance to couple and alternatively undergo further oxidation in interaction with the predominantly available oxygen species in their vicinity, restricting the catalytic selectivity. In the case of over-capacity generation of methyl radicals, however, some of the extra generated radicals reach farther away from the surface, transient to the gas phase. The relatively more difficult dissipation of the generated heat in the gas phase suggests that some of the thermally-excited methyl radicals become even more active in the area T shown in Figure 2 to undergo undesired deep oxidation reactions in interaction with the oxygen species there. This has been partially confirmed using the in-situ characterizations, where the contribution of the adsorbed oxygen and lattice oxygen over the catalyst surface on the catalytic reaction rate and selectivity have been studied [39,40]. Besides other factors, this also explains why a well-tailored rate of methane activation over the catalyst surface is desired. The optimal load and fine distribution of the active catalytic materials, in this case, correspond to the rate of methane activation fine-tuned through a balanced dynamic of sub-surface lattice oxygen generation-transfer consumption. Such balance also accounts for the impacts of operating temperature and local partial pressure of methane and oxygen. Correspondingly, the under- or over-capacity generation of methyl radicals is avoided, and coupling reactions are facilitated by combustion reactions. In fact, the desired capacity of a catalyst can be established partially by tuning the concentration of active components (e.g., manganese) in particular close to the catalyst surface, impacting the oxygen lattice transfer directly through heterolytic mechanism or indirectly through homolytic mechanism [19,40]. This is in line with other observations considering the near-surface Mn-concentration as a rate-determining factor in the activity of this catalyst [18,41].

**Figure 3:**
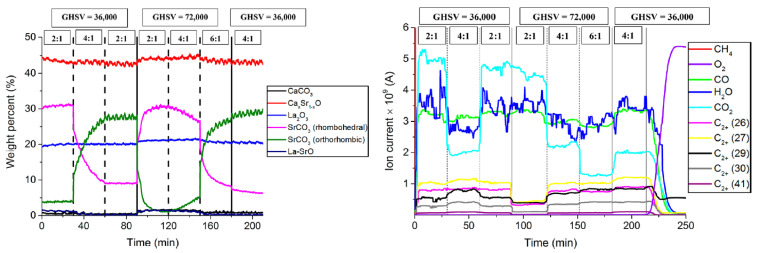
Typical recorded trends of (**Left**) operando XRD analysis of dynamic transformation of the phases over La-Sr/CaO catalyst and (**Right**) mass spectrometry measurements of the involved reactants and products under different sets of OCM reaction conditions [38].

All these phenomena over and around the catalyst surface are interconnected, and their impacts can be correlated with the characteristics of the material phases involved across the catalyst body. In particular, it tried to study the rate of generation of methyl radicals and their evolutionary selective conversion while tracking the dynamic transformation of the material phases over the catalyst surface. These have been studied using in-situ characterization techniques, including operando XRD, XPS analysis, and Raman spectroscopy [18,19,20,21,37,42,43]. In particular, operando X-ray Computed Tomography (XRD-CT) techniques have been utilized to study the dynamic transformation of the involved material phases and their corresponding roles in the catalytic reaction mechanism. Using such techniques, Mn-Na-WO_x_/SiO_2_ and LaO/CaO research benchmark catalysts have been studied under various OCM reaction atmospheres and conditions, namely the reaction temperature and methane-to-oxygen ratios [37,42,43]. For the lanthanum-based catalysts, it has been demonstrated that the transformation of La(OH)_3_, LaOOH, La_2_O_3_, and La_2_O_2_CO_3_ to each other or transformations of SrCO_3_ rhombohedral under various temperature and methane-to-oxygen ratios, could reveal important information regarding the appearance and contribution of the involved phases in the reaction mechanism [33,37,43]. In particular, the type, intensity, and stability of the formed carbonates and their significance on the OCM selective catalytic performance under various reaction conditions have been studied in correlation with thermally driven patterns of selective conversion [38]. Figure 3 shows typical trends of catalytic performance and dynamic transformation of material phases for the reference LaO-Sr/CaO catalyst under different sets of reaction conditions, namely GHSV and CH_4_/O_2_.

Figure 3 enables the tracking of the material phase transformation while varying operating parameters, such as the transformation of SrCO_3_ under various temperatures and methane-to-oxygen ratios and the monitoring of catalytic performance. Reducing GHSV and CH_4_/O_2_ increases the methane conversion, the generated heat, and the reaction temperature. This thermal effect should be analyzed along with the contribution of lattice oxygen and non-stoichiometric proportion of active components in the formation of these phases and shaping the selective performance of the tailored catalysts.

It has also been demonstrated that the basicity of some OCM catalysts could be correlated with the amount, type, and stability of carbonates formed [38]. In fact, the formation of certain stable carbonate phases of the active catalytic materials has been identified as an indicator for a selective OCM catalyst [44,45]. Having reviewed the activation mechanism and the contribution of different oxygen species in the desired and undesired reactions explained in Section 2.1, it can be assumed that the stable carbonates may play a role in partially regulating the dynamic lattice oxygen transfer between the subsurface and surface of catalysts. This can moderate the generation rates of dissociated oxygen species and methyl radicals on the surface and positively impact the catalyst selectivity.

Dynamic transformation of the involved phases under inert and reactive atmospheres has also been studied for different benchmark silica-supported Mn-Na-W-Ox catalysts [18,42,43,44,45,46,47,48]. In an attempt to study these from a mechanistic point of view, the impacts of both the physical structure and chemical composition of the catalyst should be taken into analysis. The structural characteristics of the support are mainly focused on, and the positive implications of the fine distribution of active components on their selective catalytic performance for various Mn-Na-W-Ox/SiO_2_ catalysts have been highlighted [18,42,43]. By analyzing the involved surface chemistry and the catalyst characteristics from this perspective, the Na_2_WO_4_ phase has often been identified through ex-situ characterizations [44,46] as an indication of a selective Mn-Na-WOx catalyst. However, this phase is hardly stable under the OCM reaction atmosphere. Compared to the Na_2_WO_4_ phase, the Mn_2_O_3_ phase appears to be relatively more stable under OCM reaction conditions, and its presence can be correlated with the selective catalyst performance [42]. In fact, the appearance of the material phases with higher oxidation numbers of Mn, equivalent to the availability of oxygen in their structure in over-stoichiometric proportion, could hint at regulating the lattice oxygen supply to the surface and, thereby the intensity of the surface activation [47]. The distinguished contributions of the catalytically active material phases in banking or releasing lattice oxygen in interaction with the support material should be further investigated. For instance, the role and potential of the adsorbed oxygen and the lattice oxygen species on the catalyst surface in activating the molecular reactants can also be tracked through transient responses analysis [26,27].

### 2.2. Catalytic Materials

Screening hundreds of catalysts, composed of various metal oxides, for OCM application was started by Keller and Bhasin [6]. Catalysts composed of Mn, Cd, Pb, Sn, Sb, Bi, and Tl were identified to show superior catalytic performance, while Li, Mg, Zn, Ti, In, Mo, Fe, Cr, W, Cu, Ag, Pt, Ce, V, B and Al showed little or no activity [6]. The active metals seem to exhibit a common characteristic, meaning that they can cycle between at least two oxidation states, as supported by thermodynamic calculations.

Among the reported distinguished reference catalysts’ materials, the relatively less complex Li/MgO catalyst has been extensively investigated primarily to understand the OCM catalytic mechanism, yet has generally shown an unstable catalytic performance. Relatively more complex OCM catalysts, such as Lanthanum-based catalysts [10,38,49] and Mn-based [50,51,52] catalysts, have demonstrated a relatively more stable performance. Eventually, stability and selection are the main desired characteristics of an efficient OCM catalyst with the promising perspective of utilization on an industrial scale. Nevertheless, the C_2_-selectivity of La-based and Mn-based catalysts has been reported to be mostly below 80% for the methane conversion of above 20%. However, Table 1 provides an overall view of the distinguished catalytic performance reported for selected OCM benchmark catalysts yielding above 20% C_2_H_4_. These catalysts have been prepared using different preparation methods, including supports and promotors.

Some of these data have not been explicitly provided in some of the references; rather, they were calculated based on other available data. Some others have not been provided and could not be calculated, so these were left blank. The high, medium, and low stability assigned to the reported/known stability of each catalyst are relative qualitative observations/understanding. Comprehensive reports summarizing extensive amounts of data are available elsewhere, for instance, in reference [4], where a long list of catalysts and promoters and their performances have been reported.

#### 2.2.1. Chemical Functional Analysis of the Active Catalytic Materials

The activity and reducibility potentials (i.e., being able to cycle between two or more oxidation states) of alternative alkaline earth and rare earth metal oxides in the OCM catalysts are considered the primary indicators for utilizing them in the form of an individual or solid-solution active components [4,6,10,23,40,50].

Selection of the components should also accommodate the requirements of the targeted operating strategy, namely co-feeding, oxygen-dosing, or sequential alternating feeding of methane and oxygen. For instance, in a chemical looping cycle, the time needed for the completion of the reduction of some metal-oxides (e.g., Mn and Co) in comparison to their oxidation is preferably shorter. In this case, relatively low gas-phase oxygen concentration and more selective catalytic performance (i.e., 80–90% C_2+_ selectivity) under redox mode or oxygen-dosing can be expected for some of these catalysts [50,54]. Rare earth oxides such as Ce, Nd, Sm, and Tb, on the other hand, perform well in the co-feeding mode [10] because fast reducibility is not a decisive factor there. For the catalyst chosen to operate under an oxygen-dosing strategy, for instance, in OCM membrane reactors, other factors, such as the stability and selectivity of the catalyst operating under a low-oxygen reaction atmosphere, are crucial. Analyzing the characteristics of the catalyst should even cover the aspects regarding the susceptibility to the coke deposition and possible interactions of the catalytic and membrane materials in such operation.

Besides analyzing the catalytic performances of the active components under various feeding strategies, their interaction with oxygen, water, and carbon dioxide, respectively as strong and mild oxidative agents or with other by-products and inert species, have also been discussed in the literature [61,62].

Besides the relevant characteristics of the active components, the characteristics and functionality of the promoters should also be analyzed and synchronized with the overall material-chemical characteristics of an OCM catalyst. Various promoters, co-promoters, and additives have been tested in the recipes of OCM catalysts, and their functions and promoting impacts have been extensively studied. Some of the reported data in the literature in this regard are reviewed here in light of a general understanding of the contributions of the promoters. Promoters usually stabilize the position of active metals on the OCM catalysts’ structure, prevent their migration, and improve the efficiency of electron or proton circulation between the components over the surface and in the subsurface of the catalyst body. For instance, promoting the lanthanum-oxide catalyst with strontium (Sr), as incorporated in the catalyst bulk structure, emboldens the impact of created defects and active sites hosting highly active oxygen species and strengthens their basicity. This could lead to the reduction of the number of remaining weakly adsorbed oxygen species in accordance with the discussed mechanism in Section 2.1.1. Therefore, the intensity of the undesired reactions comprising further conversion of the methyl radicals and hydrocarbons to carbon oxides will be attenuated [63]. Similarly, the effect of promoters on the performance of Mn-Na_2_WO_4_/SiO_2_ catalyst and their functional mechanisms have been investigated [64,65]. In fact, strontium-promoted sodium tungstate has a promising potential not only in terms of improving the stability of the catalyst [65] but also with regard to its electrical characteristics, which can positively affect the activation cycle and the catalytic selectivity.

Some attempts have been made to correlate the surface chemical characteristics of the OCM catalysts, especially the measured quality and quantity of its relative acidity-basicity strengths and the oxide surface reducibility potential, with the observed rates of methane conversion and C_2+_ formation [66].

The material characteristics, which facilitate the generating of thermodynamically stable carbonates and thermally stable oxide support, have shown potential for selective catalytic performance [33,44,67,68]. On the other side, the formation of oxycarbonates has been identified as an indication of the undesired chemical interaction of carbon dioxide with the catalyst [69].

The elements tested as promoters under representative OCM conditions resulting in the catalysts capable of providing more than 10% C_2+_ yield have been listed, for instance, in an early report by Bhasin and Campbell [63]. Promoting effects of Sr, Na, and W, respectively, in the research benchmark La_2_O_3_ and Mn-Na_2_WO_4_/SiO_2_ catalysts have been well studied [70,71,72]. Moreover, the impacts of their migration, leaving the catalyst under reaction conditions, have been highlighted not only on the resulting catalyst instability but also in causing a serious challenge to the reactor operation, particularly membrane reactors. Even fresh introduction of inexpensive salts such as sodium chloride to substitute the migrating components has been examined [73]. In this case, besides sodium, chlorine can act as a promoter and contribute to securing a higher ethylene-to-ethane ratio [73,74,75,76]. The observed improved ethylene selectivity while using promotors is widely believed to be due to their role in initiating the desired gas-phase dehydrogenation/oxi-dehydrogenation reactions. However, sodium and other metal promotors, which are close to their molten state under OCM reaction temperature, can intensify the activation of molecules. Depending on the applied operating conditions and the generated flux of methyl radical on the catalyst surface, this can lead to improving or degrading the selectivity, respectively, in the low- and high-reaction intensity.

Other dopants and promoters, such as Mn and Ba, have also been tested in different OCM catalysts either individually or in combination, for instance, as is the case for well-studied Mn-Na_2_WO_4_ model-catalysts. In an attempt to explain the contributions of these components in improving the catalyst’s performance, ex-situ and in-situ phase transformation in this and other OCM catalysts have been studied. For instance, it has been demonstrated that tungstate stabilizes sodium on the catalyst surface and sodium (Na+) facilitates the transformation of amorphous silica to α-cristobalite, which is one of the few phases preserving their crystalline functionality under high-temperature OCM reaction and boosting the formation of active sites [22,76,77]. By following the dynamic transformation of α-cristobalite to quartz and tridymite from one side and MnO_2_, Na_2_WO_4_, WO_4_, and Mn_2_O_3_ from the other side, the potential of selective conversion under the investigated range of operating conditions has been evaluated [44,78,79,80]. It should be highlighted, though, that the targeted manganese oxide phases cannot be easily detected in the reduced state. Nevertheless, the role of tungsten oxides in its V and VI states and manganese oxides II, III, and IV, Na-O-Mn, and Na-O-W species in connection with the quantity and quality of the oxygen lattice have been investigated under different oxidized and reduced atmospheres [44,78,79,80,81,82,83,84,85].

It should be taken into consideration that properly selected types and quantities of the promoters contribute to improving the performance of the OCM catalysts, partly through their interaction with the support. This can be analyzed mainly by following the transformation of the chemical and physical characteristics of the support during and after the OCM reaction. Hence, the contributions of dopants, promoters, and support should be discussed simultaneously.

#### 2.2.2. Material- and Structural-Based Analysis of the Catalysts

The type and the structural characteristics of the support can also have a significant impact on the selectivity and stability of OCM catalysts. The interaction of various support materials with the promoters and dopants is the key aspect in analyzing their impacts on the chemical and physical-structural characteristics of the catalysts. It should be highlighted that the resulting catalytic behaviors of a given active component over the supports with different structural characteristics, even if they are made of the same material, can be markedly different [51]. It is assumed that the structural characteristics of the support and the levels of surface free energy of the involved components determine the chance of distribution of the active components. Better distribution of the active components as such will facilitate a better distribution of defects across the catalyst. The distribution of defects is believed to be correlated with the distribution of local intensity of active oxygen species and the enhanced dynamic regeneration cycle of active sites, which ultimately contribute to establishing a finely distributed local intensity of methane activation and coupling reactions. Therefore, when a homogeneously distributed desired local rate of methyl radical generation can be established all across the catalyst, it favors the local rate of methyl coupling in competition with the undesired reactions and thereby improves the catalyst selectivity. This should be combined with applying the right sets of operating temperature and partial pressure of the reactants. At the same time, better distribution of the active sites provides relatively more homogenous distribution of the generated reaction heat, thereby avoiding local hot spots on the surface, which also contributes to improving the selectivity. These have been demonstrated, for instance, by comparing the observed characteristics and catalytic performances of Mn-Na-WOx catalytic materials impregnated on different silica support structures such as amorphous silica and SBA-15.

The latter showed a better distribution of active components and more selective performance [51]. The sol-gel-made catalyst with the same composition has also shown a homogenous distribution of the components across the catalytic structure and a selective performance [52].

It should be emphasized that analyzing the impacts of the structural characteristics of the OCM catalyst support in this way provides a simplified picture of the functionality of the OCM catalytic structure adopted for the engineering analysis reported in this paper. In fact, the actual meso-micro-scale phenomena determining the performance of an OCM catalytic structure are much more complicated and reflect the interactions of chemical-material-structural characteristics of the catalytic structure. However, the demonstrated capability of such a simplified analysis approach in explaining the observed behaviors of OCM catalytic performance via tracking the interactions of the dopants and active components with the support is encouraging.

Measurable physical characteristics of OCM catalysts, such as their electron conductivity, which is affected by the interaction of the support, promoters, and dopants, can also be tracked and correlated with their catalytic selectivity [35,85]. The intensity of ion-exchange interaction between the dopant and the support can be characterized through the generated defects and trapped electrons and correlated with the catalyst selectivity. For instance, yttrium oxide (Y_2_O_3_) supported catalyst doped with BaF_2_ has shown a significantly improved selectivity in reference to its undoped structure with much fewer structural defects and lower capability for generating oxygen species [86]. This is in line with the requirements of a desired activation mechanism explained in Section 2.1.1. Similarly, the in-situ measurement of the electron conductivity of trimetallic silica-supported catalyst having Sodium (Na), Tungsten (W), Manganese (Mn), or Cerium (Ce) species, exhibited the crystalline structures and morphologies which represent the bulk properties of a selective OCM catalyst [52,87]. It should be, however, highlighted that some dopants, such as tungstate, will, in fact, weaken the ion interaction of other dopants, like the ones with high-oxygen storage capacity (e.g., cerium) and the ion source (e.g., sodium). In this manner, by bridging the metal oxide and the ion source, the tung state will texturally contribute to securing a moderately controlled ion interaction. The promoting impact of the dopants on fine-tuning the ion interaction occurring on the catalysts’ bulk structure and their surface chemistry and, thereby, on their observed selectivity has been demonstrated [87,88].

Reaction conditions and the chemical-structural characteristics of each catalyst affect the adsorption/desorption interaction of the OCM catalyst surface with the molecule/radical species. These should also be reflected in the analysis by studying the oxygen-scrambling activity of the material, which ultimately affects the catalytic performance [89].

#### 2.2.3. Synthesis-Recipes for OCM Catalysts

Various synthesis methods have been applied to prepare different OCM catalysts, particularly benchmark catalysts. For instance, comparative performance analysis of silica-supported Mn-Na-WOx catalysts prepared by different methods has been reported [52,90]. The impacts of using different supports on the characteristics and catalytic performance of Mn-Na-WOx catalysts have also been studied [51]. The impacts of catalyst composition and synthesis methods on the characteristics and catalytic selectivity of other benchmark catalysts have also been studied [12,17,18,19,37,44,46,91,92]. Not only the catalyst synthesis recipe and method but also the procedure of its thermal treatment, for instance, the calcination temperature and its duration, can impact the OCM catalyst performance [42,43]. The calcination conditions and its procedure will impact the accessibility of the distributed active sites to the gaseous species and, thereby, the local reaction and the generated heat intensity over the catalyst surface, which ultimately affect the overall observed catalytic performance. In this context, the impacts of the applied synthesis and concentration of the active materials on the structural properties of the lanthanum catalysts (e.g., surface area) and on the primary activation of reactants and total oxidation of products have been discussed [93]. On the other hand, the synthesis method can affect the catalytic performance by forming nano-structure catalysts [61,94]. For instance, the distinguished characteristics and catalytic performances of the powder form and electrospun nanofibers composed of La-Ce and Sr-La-Ce oxides in different La, Sr, and Ce contents have been compared and discussed considering the formation of defects, the observed nano-structure and crystallinity in each case as well as their heat transfer characteristics [94]. The desired surface and bulk characteristics of an OCM catalyst should be specified and then established, preferably via a step-by-step tailoring-characterization approach. Still, so much extra work can be and should be performed in this context primarily to understand and ultimately tailor the desired stable-selective OCM catalyst.

As reviewed earlier, the intensity of the reaction across the catalyst can be tuned either by the homogenous distribution of the promoters or by changing the quantity of the active components in the catalyst recipe. Therefore, the specific catalyst composition, for instance, the recommended catalyst composition for the benchmark 1.9–2%Mn-5%Na_2_WO_4_/SiO_2_ [52], does not necessarily represent the best catalyst composition synthesized by different methods in different scales and a wide range of reaction conditions. Even the sequence of adding the promoters can impact its structural characteristics and its catalytic performance. For instance, it has been observed that micropores are formed when tungsten and sodium are implemented simultaneously, which can lead to different catalytic performances [52,91]. The application of simple characterization methods such as BET analysis is limited to proving the formation of these micropores.

#### 2.2.4. Spot-Reaction Intensity as a Measure for Tracking the Impacts of the Catalyst Characteristics on the Catalyst Selectivity

The intensity of the involved reactions can be represented through the kinetic equations quantifying the rates of the set of parallel-series gas phase catalytic reactions [7,8]. On one side, the mechanism should be able to qualitatively differentiate and track the distinguished contributions of different involved species in activation, propagation, and the secondary reactions of the intermediate radicals and molecules [19,25,26,27,28,29,30,31,32]. On the other hand, the OCM kinetic should enable explaining the impacts of the involved parameters and phenomena in relatively strengthening each of these particular steps [7,8]. This can facilitate tuning the rate and intensity of activation of oxygen and methane molecules and the locally generated/converted rates of radicals in the scale of the catalyst. As explained earlier, the uncontrolled sub-optimal (lower or higher than optimal) rate of radicals’ production-processing could ultimately lead to the formation of undesired carbon oxides. As long as the local reaction intensity (catalyst scale) can be kept in the desired range and determined in line with the catalyst characteristics, further selective conversion of methane and C_2_ yield can be secured using a proper reactor design. Hence, in order to secure an overall selective reactor performance, the intensity of the reaction should be tailored not only to the scale of the catalyst but also to the larger scale along the reactor. This can be achieved by designing a proper reactor feeding and the millimeter-centimeter scale characteristics of the catalytic bed, including its dimensions, thermal characteristics, and catalyst characteristics, as explained earlier. Proper addressing of these enables the establishment of proper temperature and concentration profiles along the catalytic bed, which are crucial for securing robust and selective reactor performance [54,61]. These will be discussed in further detail in the next section.

## 3. Catalyst Performance Analysis

Reviewing the catalyst functionality and characteristics should be combined with analyzing its performance, usually monitored in a fixed-bed reactor operation. In fact, the interactive impacts of the catalyst characteristics and the design and operating parameters of the reactor shape the thermal-reaction performance indicators. Particularly, the reaction intensity in the catalyst- and reactor scales and, thereby, the overall reactor performance are affected by the partial pressure of the species and the reaction temperature for given loads of the catalyst and processed gas. It should be highlighted that the spot temperature over the micrometer scale of the catalyst surface affects the local reaction rates, and the selectivity, as explained in Section 2, cannot be directly measured. However, the reaction temperature reflecting the temperature in the macro-scale atmosphere inside the reactor, which affects the overall methane conversion and yield of the products, can be measured via a thermocouple, preferably located inside the catalytic bed.

### 3.1. Bulk-Reaction Intensity as a Measure for Tracking the Interactive Impacts of the Operating Parameters and Dimensional Factors of the Catalytic Bed on Its Catalytic Performance

The main performance indicators, which usually can be directly monitored and recorded, are the reaction temperature and gas composition of the reactor outlet product stream. Accordingly, methane conversion, selectivities, and yields of the products can be calculated. However, the interactive impacts of the parameters (i.e., dimensions of the catalytic bed, feed flow, gas composition, temperature, etc.) can be mechanistically tracked through their impacts on the reaction intensity as a representative indicator of the reaction atmosphere.

As reviewed in Section 2.2.4, the spot-reaction intensity could explain selective catalytic performance from a mechanistic point of view. This reflects the impacts of the involved phenomena on the surface and subsurface of the catalyst and the impacts of the intrinsic catalyst characteristics, spot temperature, and spot concentration of the reactive species. The bulk-reaction intensity, on the other hand, is affected not only by the spot-reaction intensity but also by the dimensional aspects of the catalytic bed and the processed gas flow, as well as the bulk temperature and concentration profiles. Primarily, the heat transfer characteristics and thermal engineering of OCM reactors have proven to have a significant impact on their thermal-reaction performance [50,90].

An overall selective reactor performance can be secured only using a selective catalyst and a proper set of parameters to establish an optimal bulk-reaction intensity along the reactor as well as the spot-reaction intensity over the catalyst surface. The impacts of the operating parameters and the macro-scale phenomena in interaction with the phenomena on the catalyst scale determining the reactor performance are discussed in this subsection. Space-resolved operando measurements of the temperature and gas concentration along the catalytic bed have been utilized to reflect the impacts of the phenomena on the ultimate observed reactor performance. For instance, the intense undesired gas phase reaction predominantly formed by highly exothermic reactions is indicated by the recorded high temperature even before the gas heats the catalytic bed. On the other hand, a sharp temperature peak observed in the catalytic bed close to its beginning usually indicates predominantly catalytic reactions.

Feed composition, temperature, and the flow process in an OCM reactor, along with the reactor’s dimensional and operational factors, especially for the thermal control mechanism, are the design parameters to secure a robust-stable safe, and selective operation for maximum selective methane conversion, usually represented as highest C_2_- or ethylene-yield.

#### 3.1.1. Dimensional Factors: Gas Hourly Space Velocity (GHSV) Reflecting the Feed Flow and Dimensional Aspects of the Catalytic Bed

Gas Hourly Space Velocity (GHSV) represents the feed flow rate to be processed while passing over a catalytic bed of a given size. This parameter is usually reported as one of the testing conditions while reporting the catalytic reactor performance. This parameter, on the one hand, affects the catalytic performance by changing the effective residence time. On the other hand, and usually more importantly, it affects the potential for handling and transfer rate of the generated heat per a given area/volume of the bed and establishes the desired temperature profile. These could be represented by establishing the right reaction intensity across and along the reactor.

Inert gas dilution also affects the reaction intensity, which is the amount of reaction per volume of the catalytic bed per unit of time. In fact, gas dilution has a direct and an indirect impact, respectively, through the reaction mechanism and affects the thermal behavior of the system during the observed selective conversion in a packed-bed OCM catalytic reactor.

In a close to isothermal OCM reactor operation, expected in a fluidized-bed reactor, the gas dilution could improve the C_2_ yield primarily due to reducing the undesired contribution of gas phase reactions. These have been recorded in the case of using a catalyst volume fraction of the bed much lower than 50%, which is, in fact, the lower bound in a packed-bed reactor [95]. Interpreting the results of the OCM packed-bed OCM reactors in this regard due to the strong non-isothermal behavior of the system is less straightforward [9,54,95,96]. Different from the dimensional parameter, the operating parameters can be set only within the boundaries of the reactor, and they are subject to variation along the bed.

#### 3.1.2. Operating Parameter: Methane-to-Oxygen Ratio

The effect of the methane-to-oxygen ratio on the scale of the catalyst was discussed earlier in Section 2, highlighting the potential for higher catalyst selectivity due to the reduced partial pressure of oxygen. In this subsection, the impact of the established methane-to-oxygen ratio in the scale of reactors in the context of bulk-reaction intensity is discussed. A higher methane-to-oxygen ratio on the reactor scale means less available stoichiometric oxygen for converting methane, which consequently leads to lower methane conversion and ethylene yield. In fact, even using methane-to-oxygen ratio 2, more than 50% methane conversion cannot be obtained. Higher selective methane conversion and a lower risk of forming an explosive atmosphere can be secured using alternating and oxygen-dosing reactor feeding. For instance, oxygen-dosing along a membrane reactor enables the tuning of the local methane-to-oxygen ratios and the reaction intensity along the reactor.

#### 3.1.3. Operating Parameter: Reactor- and Reaction-Temperature

In academic publications, the reactor set-point temperature is usually reported along with the reactor performance indicators. This is the temperature measured and controlled inside an electrical-heater around the reactor. In fact, preferably, the reaction temperature measured inside the catalytic bed should be reported as it better represents the reaction atmosphere, which results in given reactor performance indicators. However, this is not often measured due to difficulties in the measurement of such temperature in the standard bench-scale fixed-bed reactors. Depending on the content of the inlet methane and oxygen and the extent of their conversion, a significant difference between the furnace set temperature and the actual reaction temperature inside the catalytic bed could be expected [9]. Nevertheless, the impacts of higher reaction temperature can be indirectly tracked by monitoring the ascending C_2_H_4_/C_2_H_6_ ratios.

The reaction temperature is not only affected by the reactor set temperature but also by the feed composition and the dimensional aspects of the reactor, as explained earlier. Recognizing the different factors affecting the reaction temperature and its deviation from the reactor set temperature enables better analysis of the observed reactor performance under different sets of operating parameters. Particularly, the thermal impacts of the dimensional parameters, such as the GHSV, on the reactor performance should be differentiated from the observed changes in catalytic performance directly affected by the parameters, which could be tracked through the catalytic reactions mechanism. Improving C_2_-selectivity and yield primarily due to thermal impacts of building up an intense reaction atmosphere, using a lower range of fluidization velocities, has been observed even in a fluidized-bed reactor known for its relatively mild thermal-fluctuation. Such thermal impacts are more significant in packed-bed reactors. Typical trends highlighting such distinguished impacts are presented in Section 3.1.4.

#### 3.1.4. Typical OCM Reactor Performance Affected by the Interactive Impacts of the Operating Parameters and Dimensional Factors

Much data showing the typical performance of OCM catalysts has been reported in the literature. It should be highlighted that instead of aiming at a detailed analysis of all reported information in each category, the context and the circumstances affecting the interpretations of the data are focused on in this review paper. Some reported behaviors and conclusions in the literature will be revisited here, and some facts and hard-core knowledge will be highlighted. Special attention has been devoted to analyzing the OCM reactor performance in the framework of thermal-reaction engineering and critically reviewing the reported catalytic performances in the literature from this perspective.

**Figure 4:**
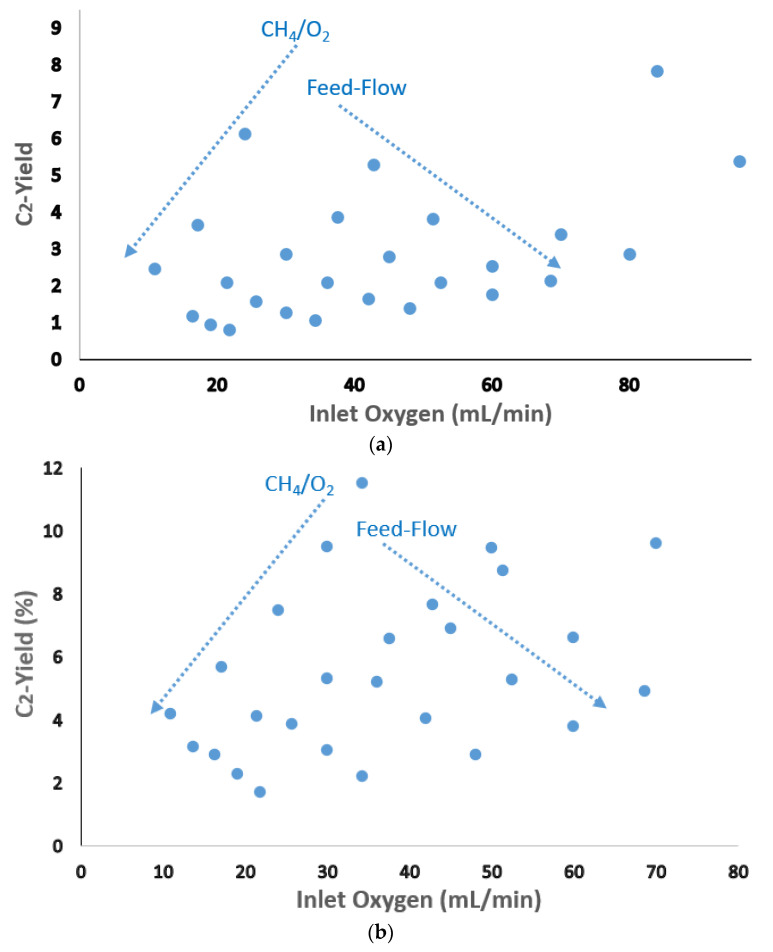

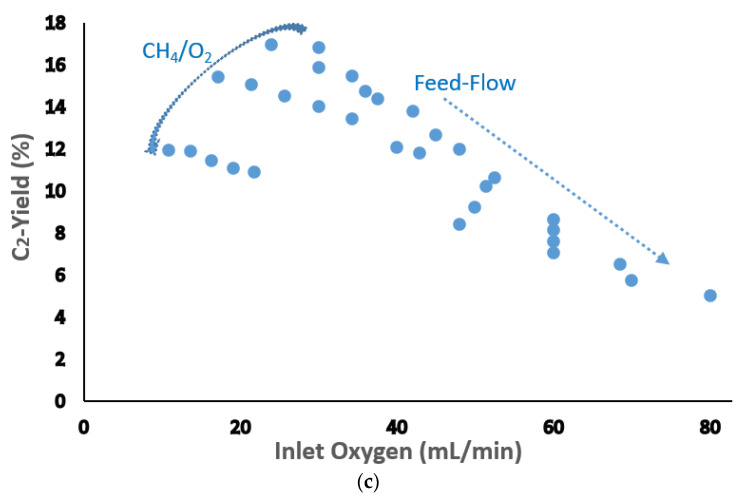
Typical experimentally recorded performance of testing OCM catalyst under wide range of operating conditions; (**a**) Top: 750 °C, (**b**) Middle: 800 °C, (**c**) Bottom: 850 °C; methane-to-oxygen ratios: 1.5, 2, 2.5, 3, 4, 6, 10; no dilution; Feed flow (cm^3^/s): 120, 150, 180, 210, 240, (raw data used to generate these graphs are available in reference [9]).

For instance, the results of extensive 630 experiments under different sets of methane-to-oxygen ratio, dilution, temperature, and GHSV have been reported for the research-benchmark Mn-NaWOx/SiO_2_ catalyst [9]. Representing some of those data in the form of trends shown in Figure 4 highlights the interactive impacts of the dimensional and operating parameters on the reactor performance.

Figure 4 shows a typical experimentally recorded C_2_ yield for a relatively low (750 °C), average (800 °C), and hot (850 °C) reactor set temperatures under a wide range of variation of methane-to-oxygen ratios and feed flow rates. The dotted arrows show the trends of variation of inlet oxygen due to increasing feed flow or increasing methane-to-oxygen ratios, highlighting the trajectory of observed C_2_ yield in each case. The correspondingly representative cold, average-hot, and hot reaction atmospheres can be distinguished by tracking the impacts of varying the inlet oxygen content. By varying the inlet oxygen content, established by varying feed flow, the observed C_2_ yield is usually reduced. The reason for this is the fact that a significant part of methane and the whole inert remains unreacted. Therefore, the interaction of the energetic reactants is less probable, leading to lower C_2_ yield, particularly in higher feed-flow, resulting in a cold reaction atmosphere. On the other hand, varying the inlet oxygen content could be established by varying methane-to-oxygen. A straightforward proportional correlation between the observed C_2_-yield and variation of oxygen inlet in this way is expected at the cold and average-cold reaction atmospheres, as seen in Figure 2 (top and middle). This indicates that there is the capacity for increasing the reaction intensity in the relatively cold and less-intense reaction atmospheres by varying methane-to-oxygen ratios so that a catalytically representative performance is obtained. Up to this extent, introducing higher oxygen in this way can lead to increasing the reaction intensity in a cold atmosphere, which is in favor of improving the C_2_-yield reactor primarily as it serves to secure the desired thermal performance. Therefore, in these relatively cold atmospheres, increasing the inlet oxygen content by reducing the methane-to-oxygen ratio while keeping the feed flow constant increases the reaction intensity and the methane conversion and, thereby, the C_2_ yield. However, as seen in Figure 4 (bottom), in the hot reaction atmosphere, C_2_ yield shows a maximum around methane-to-oxygen in the range of ratio 4–6. In this manner, increasing methane-to-oxygen ratios above ratio 4 reduces the reaction intensity methane conversion and reduces the C_2_ yield. However, reducing methane-to-oxygen ratios (e.g., ratios 1.5–3) too far can reduce C_2_ yield primarily through a significant reduction in C_2_-selectivity. Contrarily, increasing the inlet oxygen by introducing higher feed flow will always further cool down the reaction atmosphere and reduce the C_2_-selectivity and yield.

On the other hand, the reaction temperature is already high in high reactor set temperatures, and the observed reactor performance is affected primarily by catalytic performance. Varying the inlet oxygen content via reducing the inlet methane-to-oxygen ratio will increase the methane conversion. The C_2_ selectivity can, however, decrease significantly by reducing the methane-to-oxygen ratio below 4 in such a most intense extra-hot atmosphere so that the C_2_ yield shows a maximum around this range. Above that ratio, the oxygen content that is too low is not enough to secure significant methane conversion, and therefore, the C_2_ yield is reduced. This is what has also been reported under a dosing atmosphere [54] as well as close to an isothermal fluidized bed reactor [95]. Increasing the GHSV or the feed flow on one side reduces the effective contact time and affects the thermal characteristics of the reaction atmosphere on the other side. Therefore, increasing the feed flow and GHSV has a less straightforward impact on the methane conversion and C_2_ yield by varying methane-to-oxygen ratio and feed flow rates, as shown in Figure 4.

### 3.2. Practical View on Testing and Analyzing the OCM Catalytic Performance

The general design and operating features of OCM as a high-temperature exothermic catalytic system are discussed in this section to be taken into account while analyzing the impacts of the parameters on the reaction intensity inside the reactor and its performance.

#### 3.2.1. Reactor Engineering: Dimensional Factors Forging Selective Performance

First of all, it should be highlighted that depending on the purpose of the catalyst testing, the condition and the procedure of testing could be different. For instance, for a fast catalyst screening, the observed C_2_-selectivity of the catalysts is usually measured and compared under a low-to-medium range of methane conversion. Such experimentation is usually conducted in lab-scale reactors, where oxygen conversion could remain incomplete due to the implemented high GHSV—Gas Hourly Space Velocity—and methane-to-oxygen ratios. The screened catalysts could be further analyzed with regard to their best catalytic performance, which is expected to be achieved under an intense OCM reaction environment. Therefore, screened catalysts are tested in larger beds under lower methane-to-oxygen ratios and the intense reaction atmosphere, securing the highest methane conversion and C_2_-selectivity if properly designed. The observed almost complete oxygen-conversion and high ethylene-to-ethane ratios are other indicators of such a thermal-reaction intense atmosphere. In fact, in all catalytic performance tests, it is recommended that these two important indicators be measured and reported.

On the same note, the contribution of the gas phase and surface reactions should be identified. One indicator is to monitor the CO/CO_2_ ratio; its high value usually indicates the contribution of the oxidation reactions initiated or propagated in the gas phase.

In reviewing the reported data and trends in the literature, I found that some contradictory observations are not uncommon and not surprising. Depending on the analysis context and due to the differences in the dimension and scale of the reactors used for catalyst testing, in particular with regard to the thermal characteristics of the reactor setup, sometimes even contradictory conclusions have been made with regard to the impact of the parameters on the performance of the same catalyst in different reactors. Therefore, in reviewing the reported results in the literature, the analysis context and the testing conditions, as well as the specifications of the experimental setup, should be taken into consideration. For instance, the reaction atmospheres with different dimensional- or thermal characteristics can lead to different reactor performance indicators even if the same methane-to-oxygen ratio and reactor set temperature are applied. In order to analyze these in detail, the technical specifications of the equipment and its structural design, as well as the procedure for temperature control in this system, should be reviewed.

#### 3.2.2. Reactor Engineering: Thermal Management and Control

In an OCM reactor, the methane-rich feed stream should be preheated to reach the reaction unset temperature to kick off the reaction, preferably as soon as hitting the catalytic bed. The involved net exothermic reactions generate excess heat so fast that it cannot quickly escape the catalytic bed. As a result, a temperature peak is formed, usually not too deep into the catalytic bed, where the conversion of almost all inlet oxygen and a major portion of the methane has already been secured. If the reaction is too fast due to an intense reaction atmosphere facilitated by an active catalyst, high operating temperature, high oxygen partial pressure, smaller reactor diameter, and smaller catalyst particle, the peak temperature would be thinner and taller while it is formed closer to the catalytic bed entrance. This is partially due to the fact that such rapidly generated heat cannot be dissipated in such a dense and limited space, considering the system’s boundaries and the contributions of the radiation, conductive and convective heat transfer mechanisms, respectively, to be more to less dominant under such operating conditions.

In the OCM experimental reports, the reported temperature usually does not refer to the actual reaction temperature but rather to the reactor set temperature. This temperature in a lab-scale reactor could be the set temperature inside the electrical furnace, which is measured via a thermocouple located inside the furnace while its transmitted signal is used as a set point to tune the applied electrical power to the heating element accordingly. Alternatively, the external temperature of the reactor wall could be monitored and controlled. These result in different thermal management and, consequently, catalytic performance compared to the case in which the reaction temperature inside the catalytic bed is set to be controlled.

The on/off frequency of the heating/non-heating function of an electrical heater should be ideally fine-tuned while running the experiments. It is crucial to differentiate the thermal impacts driven by the internal exothermic reaction from the ones caused by external thermal control mechanisms. Besides temperature, feed flow and concentration are also controlled to establish the desired reaction intensity in the reactor.

The activation rate and the concentration of reactants converted per reactor volume reflect the distribution of reactions across and along the catalytic bed and represent the reaction intensity. Any observed catalytic performance not addressing the thermal-reaction requirements of this catalytic system to establish a desired reaction intensity over the catalyst and along the reactor could not be representative and a proper base for analysis. In fact, the review analysis reported in this paper provides further clarifications on the distinct contributions of the operating parameters and catalyst characteristics on shaping the catalytic performance via tracking the catalytic mechanism and thermal characteristics of the reaction environment. Accordingly, the requirements for securing a selective catalytic conversion were highlighted to reflect the desired catalyst characteristics to interact with the local temperature and concentration and secure an optimal reaction intensity.

## 4. Conclusions and Visions

### 4.1. Summary and Conclusions

Distinguished features of the OCM reaction mechanism were reviewed in this paper to track the impacts of the catalysts and reactor’s characteristics, as well as the operating parameters on the thermal-reaction performance of this system. In particular, tracking the indications and the impacts of the local (spot) reaction intensity, reflecting the intensity of the interaction of the catalyst surface with the active radicals and molecules, can explain the limitation of selective conversion in the catalyst scale. In addition to the type and quantity of the support and the active catalytic materials, their level of homogenous distribution over the catalytic structure also affects the reaction intensity and, thereby, the observed selectivity. It is expected that the parameters causing a sudden and intense spot-oxygen release or accumulation on the OCM catalyst surface, hinting at a relatively less controlled rate of activation of reactants and generation of methyl radicals there, can consequently lead to relatively unselective catalytic performance. The chemical-material-structural characteristics of the OCM catalysts should be ultimately tailored to tune the rate of activation of reactants and suppress the undesired reactions. This establishes the rate and intensity of activation of oxygen and methane molecules to generate methyl radicals in the desired distributed spot intensity while their excessive accumulation is restrained.

Tuning the load of reaction across and along the reactor, as well as bulk reaction intensity, is also an important aspect to be addressed using proper reactor engineering. Fine-tuning the spot-reaction intensity over the catalyst surface and bulk-reaction intensity across the reactor enables tuning the contribution of the surface reactions, radical reactions, and gas-phase reactions to shaping the overall OCM reactor performance. This requires a proper catalyst design and reactor engineering to establish the right temperature and concentration profiles across the reactor. These aspects need to be considered when interpreting the reported reactor performance indicators and results of catalyst testing.

The impacts of the dimensional design aspects of the catalytic bed and the processed feed flow, along with operating temperature and feed composition in terms of methane-to-oxygen ratio and diluent on the performance of the OCM reactor, were discussed primarily in the context of thermal-reaction engineering of this system. The impacts of the local temperature and the gas composition over the catalyst surface can be tracked and connected to the selective performance of the catalyst through the reaction mechanism and the increase or decrease of the spot rates of generation or consumption of the species.

### 4.2. Vision: Priorities for Future Research and Development

In the catalyst scale, identifying the main desired characteristics that can secure a stable and selective catalytic performance remains the main aim of catalyst characterization-tailoring. Efficient characterization techniques such as operando-XRD and Raman spectroscopy should be part of the research strategy to track the transformations of the involved phases and species as well as the hierarchical cause/effects of the related phenomena. This enables the identification of their roles in the reaction mechanism and, accordingly, tailoring the desired material-structural characteristics of the OCM catalyst. In particular, in-situ and operando-electron conductivity has the potential to provide valuable information in this regard. In such a research roadmap, the in-situ monitored trends of phase transformations, including the formation of material phases such as carbonates, which could be studied and possibly correlated with the intensity of surface reactions and the catalyst selectivity. Particularly, the contribution of the appeared phases in moderating the activation rates and suppressing the undesired oxidation reactions via tuning the rate of lattice oxygen generation-transfer-consumption cycle should be studied. The key issue is that if a significant adsorbed oxygen remains on the catalyst surface, it causes a nonselective catalytic performance. Using a catalyst with proper chemical-structural characteristics under the right set of operating conditions, minimum adsorbed oxygen remains on the surface as oxygen is activated proportional to the potential of the catalyst in establishing a balanced lattice-oxygen generation-transfer-termination contributing to selective conversion.

In the ultimate attempts to synchronize the impacts of parameters affecting the phenomena on the catalyst- and reactor scales and, thereby, the overall systems’ selective performance, their interactive impacts should be thoroughly studied. This could become a part of the deep screening of catalysts, for instance, by combining in-situ XRD or electron conductivity measurements with high throughput catalyst screening. The extracted knowledge could be useful even for the design of reactors, such as membrane reactors, with higher potentials for tuning the local parameters along the catalytic bed.

Similar approaches could be adopted and applied for the OCM catalysts capable of operating at low temperatures and non-atmospheric operations as they are preferred for industrial applications, considering their practical prospects of more efficient operation.

Nevertheless, a catalytically facilitated relatively low-temperature OCM operation is preferred mainly for securing a selective, safe, robust, and thermally controllable operation. Higher temperatures increase the risk of the build-up of an explosive atmosphere and a poorly controlled thermal-reaction performance. These are the most serious safety concerns regarding an OCM reactor operation, which are emboldened, especially when the feed streams with lower methane-to-oxygen ratios are processed. Establishing the desired catalyst characteristics to secure a controlled activation of the oxygen and methane molecules in a lower temperature range cannot only address these but also favor the coupling reaction.

Similarly, setting the operating pressure in an OCM reactor impacts not only the catalytic performance through the gas-catalytic reaction mechanism but also the energy efficiency of the whole process. In this regard, selecting the operating pressure is an engineering decision and should be synchronized with the design-operation requirements of the industrial-scale reactor and process. The impacts of pressure on the reaction mechanism can be tracked directly through the partial pressure of the species and indirectly through affecting the thermal characteristics of the reaction atmosphere, which are reflected in the reaction intensity per volume of the catalytic bed. Since the majority of the OCM studies have been conducted in atmospheric pressure or slightly above it, this review paper solely reflected the observations in those studies.

## Figures and Tables

**Table 1:** Overall view on the distinguished catalytic performance (higher than 20% C_2_H_4_-yield) of selected benchmark catalysts reported for oxidative coupling of methane highlighting the impacts of different preparation methods, supports, and promotors.

Catalyst Benchmark-Type	Preparation Method	Reactor Type	T (°C), CH_4_/O_2_	GHSV (h−1)	XCH_4_ (%)	SC2+ (%)	YC_2_H_4_ (%)	C_2_H_4_/C_2_H_6_	Relative Stability	Ref.
Mn-Na-WOX/SiO_2_	Impregnation	Fixed-bed Reactor	800, 4	2700	37	66	20.2	5	Medium	[53]
Mn-Na-WOX/SiO_2_	Impregnation	Membrane Reactor	820, 2.5	2000	39	66	20.3	3.9	Medium	[54]
Mn-Na-WOX-SiO_2_	Sol-gel	Membrane Reactor	850, 3	2000	31	78	20	4.6	Low	[52]
Mn-Na-Ce-WOX/TiO_2_	Impregnation	Fixed-bed Reactor	775, 2	4800	46	57	21.1	4.1	-	[55]
La-PrOx-Li-COx	Impregnation	Chemical Looping	700, -	180	43	70	20	3.3	High	[56]
Li-W-Mn/MgO	Impregnation	Chemical Looping	850, -	2400	50	58	23	5.5	High	[57]
Li/MgO	-	Fixed-bed Reactor	770, 3	655	59	53	21.2	2.1	Low	[58]
Li-S/ZrO2	Impregnation	Fixed-bed Reactor	800, 2	6000	43	79	25.2	2.8	-	[59]
Li-Mn	-	Fixed-bed Reactor	760, 2.6	5540	41	68	27.9	-	-	[60]
